# Antibacterial Activity of Linezolid against Gram-Negative Bacteria: Utilization of ε-Poly-l-Lysine Capped Silica Xerogel as an Activating Carrier

**DOI:** 10.3390/pharmaceutics12111126

**Published:** 2020-11-21

**Authors:** Gulcihan Guzel Kaya, Serena Medaglia, Vicente Candela-Noguera, María Ángeles Tormo-Mas, María Dolores Marcos, Elena Aznar, Huseyin Deveci, Ramón Martínez-Máñez

**Affiliations:** 1 Department of Chemical Engineering, Konya Technical University, Ardıçlı Mah. Rauf Orbay Cad, Selçuklu, 42250 Konya, Turkey; ggkaya@ktun.edu.tr (G.G.K.); hdeveci@ktun.edu.tr (H.D.); 2 Instituto Interuniversitario de Investigación de Reconocimiento Molecular y Desarrollo Tecnológico, Universitat Politècnica de València, Universitat de València, Camino de Vera s/n, 46022 Valencia, Spain; sermed@idm.upv.es (S.M.); vicanno@etsid.upv.es (V.C.-N.); mmarcos@qim.upv.es (M.D.M.); 3 CIBER de Bioingeniería, Biomateriales y Nanomedicina (CIBER-BBN), 46022 Valencia, Spain; 4 Grupo Acreditado de Infección Grave, Instituto de Investigación Sanitaria La Fe, Av. Fernando Abril Martorell, 46026 Valencia, Spain; tormo_man@iislafe.es; 5 Unidad Mixta de Investigación en Nanomedicina y Sensores, Universitat Politècnica de València, Instituto de Investigación Sanitaria La Fe, Av. Fernando Abril Martorell, 46026 Valencia, Spain; 6 Unidad Mixta UPV-CIPF de Investigación en Mecanismos de Enfermedades y Nanomedicina, Universitat Politècnica de València, Centro de Investigación Príncipe Felipe, C/Eduardo Primo Yúfera 3, 46100 Valencia, Spain

**Keywords:** antibacterial activity, linezolid, mesoporous material, silica xerogel, ε-poly-l-lysine

## Abstract

In recent times, many approaches have been developed against drug resistant Gram-negative bacteria. However, low-cost high effective materials which could broaden the spectrum of antibiotics are still needed. In this study, enhancement of linezolid spectrum, normally active against Gram-positive bacteria, was aimed for Gram-negative bacteria growth inhibition. For this purpose, a silica xerogel prepared from a low-cost precursor is used as a drug carrier owing to the advantages of its mesoporous structure, suitable pore and particle size and ultralow density. The silica xerogel is loaded with linezolid and capped with ε-poly-l-lysine. The developed nano-formulation shows a marked antibacterial activity against to *Escherichia coli*, *Pseudomonas aeruginosa* and *Staphylococcus aureus.* In comparison to free linezolid and ε-poly-l-lysine, the material demonstrates a synergistic effect on killing for the three tested bacteria. The results show that silica xerogels can be used as a potential drug carrier and activity enhancer. This strategy could provide the improvement of antibacterial activity spectrum of antibacterial agents like linezolid and could represent a powerful alternative to overcome antibiotic resistance in a near future.

## 1. Introduction

In September 2017, the World of Health Organization (WHO) reported that Gram-negative and Gram-positive bacteria had increased their resistance to many widely used antibacterial agents. Especially, Gram-negative bacteria such as *Escherichia coli* or *Pseudomonas aeruginosa* have exhibited a drug resistance >50%, which represents a serious concern all around the world [1,2]. To overcome this problem, many approaches have been developed in recent years. Researchers have focused on the production of new antibacterial agents; however, drug manufacturing companies have given short chance to new antibacterial drugs due to the high-cost and long period of production process (up to $2.6 billion and 10 years) [3]. As an alternative, promising strategies such as improving antibacterial activity of antibacterial agents in combination with additives (e.g., Ag and Au nanoparticles, TiO_2_, CuO and ZnO) [4], broadening the antibacterial spectrum of known antibacterial drugs [5], modification or encapsulation of the agents [6], combination of dugs [7] and the design of the effective drug carrier systems [8] have attracted great interest.

Gram-negative and Gram-positive bacteria exhibit some differences in the thickness of peptidoglycan layer of cell wall and membrane structure [9]. Thickness of peptidoglycan layer in Gram-positive bacteria is about 30 nm, while Gram-negative bacteria have a thinner peptidoglycan layer. In contrast, Gram-negative bacteria include an extra outer membrane which consists of lipopolysaccharides and make them resistant against various antibacterial agents [10]. Taking these differences into account, the outer membrane of the bacteria is one of the most significant parameters that has to be considered in the design of new strategies to enhance antibacterial activity. From this point of view, the use of antibacterial drugs, which normally only present activity against Gram-positive bacteria, after the elimination of the barrier effect of their outer membrane has become a popular approach for Gram-negative bacteria killing [11,12].

Over the last decades, mesoporous silica-based materials have been applied to different areas such as carriers for drug delivery [13,14,15,16], in tissue engineering [17,18], as medical implantable devices [19], in communication protocols [20,21,22], in biosensing [23,24,25] and as antibacterial carriers [26]. Among them, silica aerogels are attractive silica-based materials for biotechnological applications with advantages such as high specific surface area and porosity, controllable pore structure, chemical stability and biocompatibility [27]. Silica aerogels are generally synthesized using conventional silicon precursors by sol-gel method under supercritical drying conditions. Nowadays, utilization of inexpensive silica precursors such as industrial by-products, inorganic and organic waste and agricultural residues, instead of conventional silicon alkoxides which are expensive [28] has attracted attention. In addition, supercritical drying is a relatively expensive and risky process due to the requirement of high pressure and temperature. For this reason, drying in ambient pressure has become popular for large scale productions in which the final material is termed “silica xerogel” [29,30]. Among possible silica precursors, volcanic tuff is an inexpensive and abundant inorganic material which is rich in silicon. Volcanic tuff is produced from aggregation of fragmented pyroclastic materials and ashes from volcanic eruptions [31]. In spite of volcanic tuff is already used in some applications such as construction (cement and concrete) and adsorption, potential applications of the volcanic tuff can be extended leading not only a decrease in its accumulation but also production of new low-cost materials [32,33]. There are seminal works in the literature about the use of silica aerogels for antibacterial applications [34,35,36]; however, the use of low-cost silica precursors to develop such relevant application has not been described until now.

**Scheme 1:**
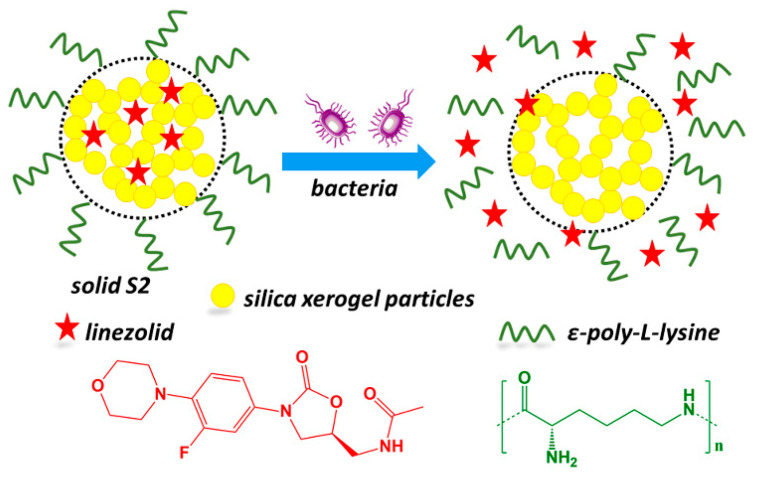
Representation of carrier system and its behavior against the Gram-negative bacteria.

In the light of the existing studies in the literature, within this work, the typically Gram-positive antibacterial activity of linezolid was aimed to be expanded against Gram-negative bacteria (*E. coli* and *P. aeruginosa*) using ε-poly-l-lysine capped silica xerogel as linezolid carrier. Additionally, as far as we know, this is the first time that silica xerogels obtained from volcanic tuff are used in an antibacterial application. The schematic representation of the carrier system and its behavior against Gram-negative bacteria are shown in Scheme 1. In the presence of bacteria, linezolid-loaded ε-poly-l-lysine-capped silica xerogel would permeate the outer bacteria membrane (due to ε-poly-l-lysine) and will allow a further penetration in the bacteria of the released antibiotic linezolid with the subsequent bacteria killing.

## 2. Materials and Methods

### 2.1. Materials and Culture Media

Volcanic tuff (SiO_2_ 71.5%, Al_2_O_3_ 14.0%, Na_2_O 4.8%, K_2_O 4.2%, Fe_2_O_3_ 2.8%, CaO 1.3%, TiO_2_ 0.5%, MgO 0.5%, P_2_O_5_ 0.3% and MnO 0.1%) was obtained from Kompass (Kayseri, Turkey). For silica xerogel synthesis, sodium hydroxide (NaOH) and hydrochloric acid (HCl) were supplied from Sigma-Aldrich (St. Louis, MO, USA). Isopropanol (C_3_H_8_O) and *n*-hexane (C_6_H_14_) were purchased from Merck (Darmstadt, Germany).

For antibacterial activity experiments, linezolid (C_16_H_20_FN_3_O_4_, MedChemExpress) was used as antibacterial agent and ε-poly-l-lysine ((C_6_H_12_N_2_O)_n_) was supplied from Chengdu Jinkai Biology Engineering Co. Ltd. (Chengdu, China). Mueller-Hinton broth and agar were obtained from VWR International (Radnor, PA, USA). Phosphate buffer saline from GenoChem World (Valencia, Spain) with pH 7.4 was used. All chemicals were used without further purification.

### 2.2. Preparation of Silica Xerogel Using Volcanic Tuff (Solid S0)

Synthesis procedure of silica xerogel consisted of three steps after pretreatment of the volcanic tuff [37]. The tuff was washed with 3 M HCl solution for 2 h at 60 °C to eliminate undesirable minerals. The treated tuff was dried in an oven at 70 °C following slurry washing until pH 7. In the first step, sodium silicate (Na_2_SiO_3_) solution was obtained with mixing the tuff and 3 M NaOH solution (1:6, *w:v*) for 5 h at boiling temperature. Gel formation was completed with the addition of 3 M HCl to the Na_2_SiO_3_ solution (~pH 10). In the second step, elimination of Na^+^ ions was conducted through distilled water washing (pH 7). Solvent exchange was provided with aging the silica gel with the water-isopropanol mixture (1:1, *v:v*) for 1 day at 50 °C. Silica network was more strengthened with immersing it into pure isopropanol for 1 day at 50 °C. Subsequently, water was removed from the silica network by washing with *n*-hexane. Finally, the gel was dried under ambient pressure drying in an oven at 50 °C.

### 2.3. Synthesis of Linezolid Loaded Silica Xerogel (Solid S1) and ε-Poly-l-Lysine Capped Linezolid Loaded Silica Xerogel (Solid S2)

At room temperature, 40 mg silica xerogel was dispersed in 20 mL of distilled water by ultrasonication for 10 min. After adding 20 mg of linezolid, the suspension was stirred for 1 day. The linezolid loaded material (solid S1) was filtered and dried under vacuum at room temperature. 25 mg of solid S1 was dispersed in 5 mL of distilled water by ultrasonication at room temperature for 10 min. Then, 50 mg of ε-poly-l-lysine was added to the suspension to cover the solid S1 surface and stirred for 1 day. The ε-poly-l-lysine capped material was vacuum filtered and washed with distilled water three times (5 mL). The final solid S2 was dried under vacuum at room temperature. Additionally, only for comparison purposes, ε-poly-l-lysine capped silica xerogel without linezolid loading was synthesized using the same procedure (solid S3).

### 2.4. Material Characterization

Powder X-ray diffraction (XRD) data were recorded with Bruker D8 Advance diffractometer (Bruker Corporation, Billerica, MA, USA) using Cu Kα radiation. Infrared spectra of the materials were obtained from Fourier transform infrared (FTIR) measurements by Bruker Tensor 27 spectrometer (Bruker Corporation, Billerica, MA, USA). Transmission electron microscopy (TEM) was performed on a JEOL JEM 2100 UHR (JEOL Europe SAS, Croissysur-Seine, France) and used to analyze morphological structure of the silica xerogel dispersed in ethanol. N_2_ adsorption-desorption measurement was conducted with a Micromeritics Tristar II 3020 surface analyzer (Micromeritics Instrument Corporation, Norcross, GA, USA). Determination of specific surface area of the silica xerogel was carried out utilizing Brunauer-Emmett-Teller (BET) method at P/P_0_ < 1.0. Pore structure including pore volume and size of the silica xerogel was investigated with data derived from Barrett-Joyner-Halenda (BJH) method. Particle size and surface charge of the materials dispersed in distilled water (1:1, *w:v*) were specified with Malvern Zetasizer Nano equipment (Malvern Panalytical, Worcestershire, UK). Density of the silica xerogel was specified by dividing silica xerogel mass to its volume. UV-visible spectroscopy carried on a Perkin-Elmer Lambda 35 UV/Visible Spectrometer (PerkinElmer Inc., Waltham, MA, USA) and thermogravimetric analyses (TGA) perfomed using a 851e Mettler Toledo balance (Mettler Toledo Inc., Schwarzenbach, Switzerland) were carried out to specify linezolid and ε-poly-l-lysine content of the materials.

### 2.5. Antibacterial Activity

Bacteria used in this study were *E. coli* ATCC 25922, *P. aeruginosa* ATCC 15442 and *Staphylococcus aureus* V329. Bacteria were grown in Mueller-Hinton agar at 37 °C 24 h. To obtain the inoculum 0.5 McFarland solution was prepared in phosphate buffer saline (PBS) and diluted to a concentration of 5 × 10^6^ CFU/mL.

In antibacterial activity studies, colony forming units (CFU) count method was used to estimate bacterial cell number. Different solid S1 and S2 concentrations were prepared diluting from the suspension of 1 mg sample and 1 mL of PBS 0.01 M solution. A 50 μL bacteria inoculum (5 × 10^6^ CFU mL^−1^) was added to each 450 μL solid suspension. Additionally, a control was prepared without solids to specify number of cell growth. Following the incubation of all samples at 37 °C for 24 h, 100 μL of each sample was dropped into a Mueller-Hinton agar plate. After incubation for 1 day at 37 °C, the number of grown colonies was counted and viability (%) was determined for each bacteria. The same procedure was carried out for the free linezolid and ε-poly-l-lysine.

## 3. Results and Discussion

In the scope of this study, the silica xerogel was synthesized by a sol-gel method through a gelation of extracted sodium silicate from volcanic tuff and then, aging and drying the gel under ambient pressure (solid S0). Solid S0 was loaded with linezolid by a diffusion process in aqueous media (solid S1) and the loaded material was capped with the cationic polymer ε-poly-l-lysine by electrostatic interaction with the negatively charged silanol groups in the material to obtain the final solid S2. Table 1 compiles the obtained materials.

**Figure 1:**
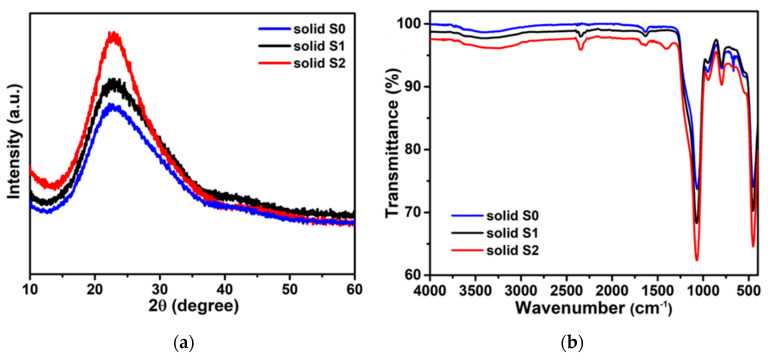
(**a**) Powder X-ray diffraction (XRD) and (**b**) Fourier transform infrared (FTIR) spectra of silica xerogel (S0), silica xerogel loaded with linezolid (S1) and silica xerogel loaded with linezolid and covered with ε-poly-l-lysine (S2).

The prepared solids were first characterized. Powder X-ray diffraction (XRD) patterns of the materials are shown in Figure 1a. The characteristic diffraction peak of amorphous silica typically found in xerogels was observed for solid S0 at about 2θ = 22° [38]. The diffractogram did not show peaks which could be related with the presence of NaCl due to insufficient washing step in synthesis. The presence of sodium ions in the silica network, could induce pore collapse due to the high surface tension of the material [39]. Thus, completely removal of Na^+^ ions is an important step to obtain appropriate textural properties of the silica xerogels. From XRD data, it can be concluded that solid S0 was successfully synthesized from the volcanic tuff without impurities. After linezolid loading (solid S1) and ε-poly-l-lysine capping (solid S2), no crystalline phases were observed in the XRD patterns which is in good agreement with literature studies [40]. Both solid S1 and solid S2 showed the same broad peak at about 2θ = 22° which indicates an amorphous silicon oxide structure.

Materials were also characterized by FTIR as shown in Figure 1b. Solid S0 showed main peaks at 1066 cm^−1^, 796 cm^−1^ and 451 cm^−1^ related to Si-O-Si asymmetric stretching, Si-O-Si symmetric stretching and Si-O-Si bending vibrations, respectively [41]. The peaks attributed to Si-OH stretching vibrations were found at about 667 cm^−1^ and 946 cm^−1^, respectively. Depending on deformation vibrations of adsorbed water molecules, the broad band centered at 3390 cm^−1^ was observed in addition to a peak at 1634 cm^−1^ [42]. In the loaded solid S1, a small increase in the intensity of the peak at 1645 cm^−1^ was determined related with N-H bending vibrations of linezolid [43]. Finally, the peak attributed to Si-OH stretching vibrations of silica network disappeared at 667 cm^−1^, probably due to the high linezolid loading. After capping with ε-poly-l-lysine (solid S2), a small shift in the broad peak centered at 3290 cm^−1^ was found due to the contribution of the vibrations of the primary amine peaks of ε-poly-l-lysine to the water band [44]. Also the appearance of a peak at 1400 cm^−1^ originated from ε-poly-l-lysine alkyl groups and an increase in the intensity of the peak at 1636 cm^−1^ was observed [45]. There were no specific peaks derived from chemical reactions that was indication of the successful capping of S1 to obtain S2 only using electrostatic interactions with ε-poly-l-lysine [46].

**Figure 2:**
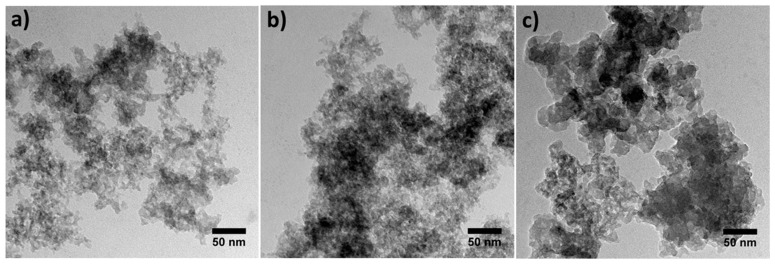
Transmission electron microscopy (TEM) image of (**a**) silica xerogel (S0), (**b**) silica xerogel loaded with linezolid (S1) and (**c**) silica xerogel loaded with linezolid and covered with ε-poly-l-lysine (S2).

TEM images of prepared materials are shown in Figure 2. It is clearly seen that S0 exhibited a typical pearl-necklace morphology (Figure 2a) [47]. A highly porous silica network with interlinked units was obtained that makes the silica xerogels desirable materials for many applications which require lightness, adsorption/desorption ability and high loading capacity [48,49]. Almost the same morphology was observed for solid S1 as in the TEM image of solid S0 (Figure 2b). In contrast, in solid S2 micrographs the formation of aggregates due to ε-poly-l-lysine capping was observed, which resulted in an increased particle size of the material (Figure 2c).

The textural properties of the solid S0 are given in Table 2. The specific surface area of the solid S0 was 195 m^2^ g^−1^. Pore volume and average pore size of the solid S0 were determined as 0.50 cm^3^ g^−1^ and 10 nm, respectively. According to pore size definition of IUPAC, the materials are classified as microporous (<2 nm), mesoporous (between 2 and 50 nm) and macroporous (>50 nm) [50]. In the light of this information, the solid S0 synthesized from volcanic tuff was classified as a mesoporous material.

As shown in Table 2, bulk density of the solid S0 was ultralow (0.037 g cm^−3^). In spite of ambient pressure drying in which gel shrinkage is not completely eliminated due to capillary stresses, solid S0 synthesized from volcanic tuff showed lower density than many silica xerogels, even aerogels synthesized from conventional precursors. As known, the selected aging solvent significantly affects density of the final materials as a result of different vapor pressure and chemical structure of the solvent [51]. In the sorting of isopropanol < methanol < ethanol < butanol < hexanol, the density of silica based material generally decreases in relation to chain length of the solvent [52]. In the present study, the use of isopropanol as aging solvent allows an effective solvent exchange with an associated decrease of gel shrinkage which confers low density to solid S0.

**Figure 3:**
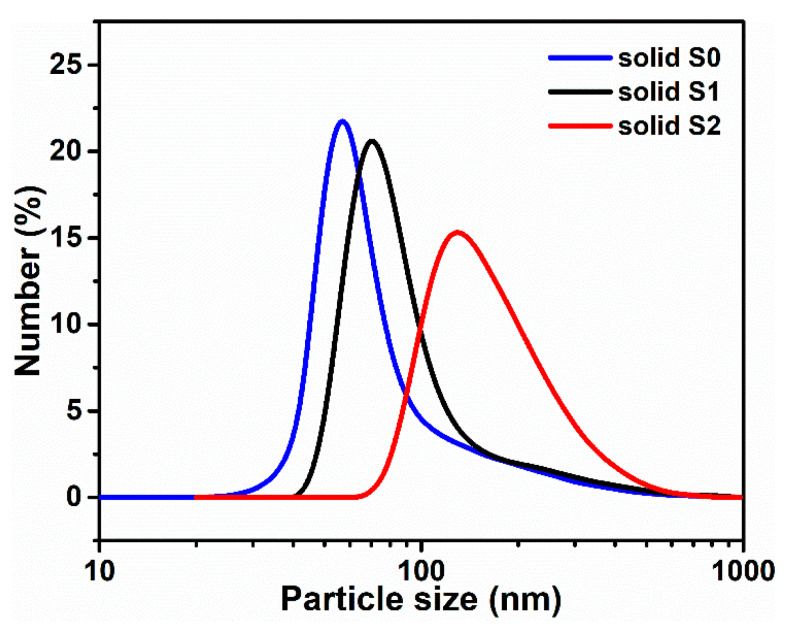
Particle size distributions of silica xerogel (S0), silica xerogel loaded with linezolid (S1) and silica xerogel loaded with linezolid and covered with ε-poly-l-lysine (S2).

Particle size distribution of the prepared materials are shown in Figure 3. As it can be appreciated, solid S0 interlinked network consisted of particles with an average size of 86 nm. In the case of linezolid loaded material, mean particle size was 95 nm which was close to that of solid S0. However, ε-poly-l-lysine capping caused an increase in the particle size of the material. Thus, S2 has an average size of 175 nm with a broad size distribution in contrast to solid S0 and S1, which is consistent with a ε-poly-l-lysine coating layer on the surface of the material [53].

The surface properties are most significant in carrier materials and the zeta potential easily describes the surface property of electrostatically stabilized materials in aqueous solutions [54]. As shown in Table 3, zeta potential of solid S0 was −46.1 mV which confirmed the high negatively charged surface of the silica xerogel due to the deprotonation of Si-OH groups on the silica surface [55]. This negatively charged state facilitates an effective surface capping with cationic compounds such as ε-poly-l-lysine. Zeta potential of the solid S1 (−42.0 mV), which is loaded with the neutral molecule linezolid, was nearly the same of solid S0. However, the zeta potential of solid S2 was 16.9 mV which indicated a significantly positively charged surface and confirmed that ε-poly-l-lysine capping was successfully carried out. Note that in the case of antibacterial activity against Gram-negative bacteria, a positively charged surface of the carrier enhances bacterial adhesion [56]. Also, other surface characteristics such as surface roughness and hydrophobicity can influence affect bacteria adhesion to the surface. Rough surfaces are favorable for bacterial attachment in contrast to smooth surfaces. Silica xerogels have hydrophilic surface that promotes bacteria growth; however, hydrophobization of silica xerogel surface with different surface modification methods can decrease bacterial adhesion [57,58].

Finally, organic contents of solids S1 and S2 are given in Table 4. Linezolid content in solid S1 (0.188 mmol g^−1^) was considerably similar to that of solid S2 (0.187 mmol g^−1^) revealing that there was no obvious linezolid release during ε-poly-l-lysine capping. Additionally, ε-poly-l-lysine content in S2 was in good agreement with other literature studies related with mesoporous silica based materials [10,12].

Once physiochemically characterized, the antibacterial activity of materials against the Gram-negative bacteria *E. coli* and *P. aeruginosa* and the Gram-positive bacterium *S. aureus* was tested by viability assays. Different concentrations of solids S1 and S2 were prepared from a suspension of 1 mg of solid in 1 mL of PBS 0.01 M. In addition, a control without solid to specify number of cell growth and a solid without linezolid but capped with ε-poly-l-lysine (solid S3, ε-poly-l-lysine content 0.08 mmol g^−1^) were also used in the studies. The materials were incubated with the corresponding bacteria (5 × 10^5^ CFU mL^−1^) for 24 h. Then, 100 μL of each sample and ten-fold dilutions were seeded in different agar plates and incubated for 1 day at 37 °C. After the incubation period, colony forming units (CFU) were counted and the corresponding viability (%) was determined. The same procedure was applied for free linezolid and ε-poly-l-lysine compounds to compare their antimicrobial activity with the action of the prepared materials. From the literature, it is known that mesoporous silica based materials have no antibacterial activity [59] and linezolid is an oxazolidinone that shows good activity to only Gram-positive bacteria [60], which is in agreement with our observations.

First, the bactericidal activity of free ε-poly-l-lysine and linezolid were studied for *E. coli*, *P. aeruginosa* and *S. aureus*. Table 5 shows the amount of free compound able to reduce until 50% the viability of the bacteria growth. In accordance with previous studies, linezolid showed activity only against the Gram-positive bacteria *S. aureus* while ε-poly-l-lysine showed a similar activity against the three bacteria.

**Figure 4:**
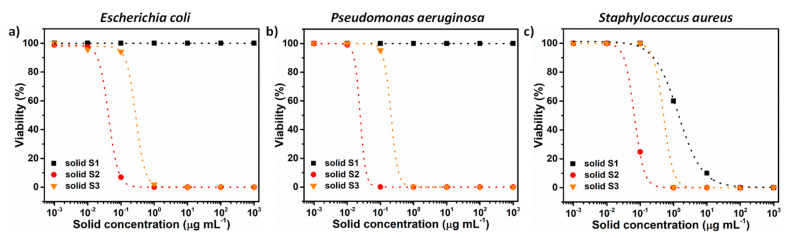
Cell viability of (**a**) *E. coli*, (**b**) *P. aeruginosa* and (**c**) *S. aureus* 24 h after treatment with solids S1, S2 and S3.

In a subsequent step, the bactericidal activity of solids S1, S2 and S3 against *E. coli*, *P. aeruginosa* and *S. aureus* was studied in the same conditions. Results are shown in Figure 4. As expected, the combination of silica xerogel and linezolid in solid S1 was unable to inhibit Gram-negative *E. coli* and *P. aeruginosa* growth and showed some activity when tested against the Gram-positive *S. aureus*. Solid S3 which only contains ε-poly-l-lysine, displayed a certain inhibition of the three bacteria growth. The most remarkable behavior is found for solid S2 which showed a synergistic antibacterial activity against the three studied bacteria. Enhancement of the toxicity against Gram-negative bacteria is attributed to the interaction of the positively charged particles S2 to the bacteria, which induced displacement of the ε-poly-l-lysine cap and release of the entrapped linezolid. In addition, ε-poly-l-lysine induced bacterial wall damage, allowing linezolid to gain access into the cell and enhancing toxicity [61]. Table 6, gathers the amount of each tested solid able to reduce until 50% the viability of the bacteria growth.

**Figure 5:**
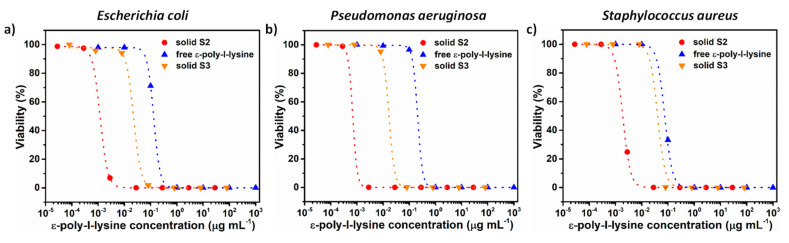
Comparison of viability of (**a**) *E. coli*, (**b**) *P. aeruginosa* and (**c**) *S. aureus* 24 h after treatment with ε-poly-l-lysine, solids S2 and S3 in terms of equivalent ε-poly-l-lysine concentration.

**Figure 6:**
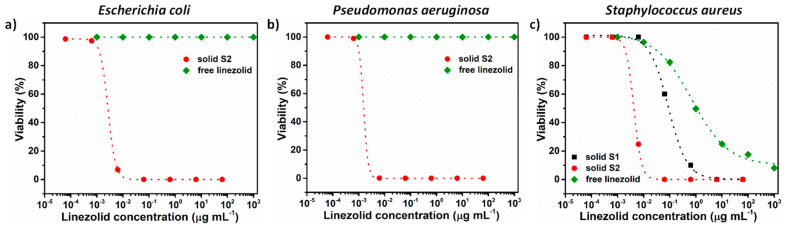
Comparison of viability of (**a**) *E. coli*, (**b**) *P. aeruginosa* and (**c**) *S. aureus* 24 h after treatment with linezolid, solids S1 (only for *S. aureus*) and S2 in terms of equivalent linezolid concentration.

To confirm the obtained results, viability of solid S2 was represented as a function of the amount of ε-poly-l-lysine (Figure 5a–c) and linezolid (Figure 6a–c) present in the material and compared with the viability of the corresponding free compound. It can be clearly seen that ε-poly-l-lysine played an important role in inhibition of bacteria growth. 0.13, 0.209 and 0.0767 μg mL^−1^ of free ε-poly-l-lysine was needed to kill 50% of *E. coli*, *P. aeruginosa* and *S. aureus*, respectively. However, nanoformulation of ε-poly-l-lysine as in solid S3 resulted in a 6, 12 and 2-fold decrease in the amount of the active compound needed to obtain the same bactericidal effect in *E. coli*, *P. aeruginosa* and *S. aureus*, respectively. It is known that antibacterial activity of ε-poly-l-lysine depends on its conformation which is related with parameters such as temperature, pH or chain length [62]. Probably, nanoformulation of ε-poly-l-lysine contributes to a more expanded conformation with an enhanced exposition of its α-amino groups with a consequent increase of antibacterial activity [63]. Likewise, the best activity is found for solid S2, were 114-, 311- and 40-fold lower amount of ε-poly-l-lysine was used to obtain the same effect in *E. coli*, *P. aeruginosa* and *S. aureus*, respectively. In terms of linezolid concentration (Figure 6a–c), free linezolid is not toxic for the Gram-negative bacteria of *E. coli* and *P. aeruginosa*, whereas it highly contributes to obtain an enhanced toxicity when incorporated in S2. For *S. aureus*, linezolid nanoformulation (solid S1) results in a 11-fold decrease in the concentration of antibiotic needed to obtain a viability of 50%. Even more, solid S2 contains a 246 times lower amount of the active compound to achieve the same results, which confirms the great effectivity of the final formulation S2.

As a result, it can be concluded that the antibacterial activity of S2 was highly better than that of the free linezolid and ε-poly-l-lysine for the three studied bacteria and opens the possibility of using the Gram-positive active antibiotic linezolid against Gram-negative bacteria such as *E. coli* and *P. aeruginosa*.

Combination of well-known antibacterial compounds in new synergic nanoformulations could represent a new promising approach to handle the increasing bacterial resistance to conventional antibiotics. Methodologies as the developed in the present work results in the co-delivery of antibiotics which could achieve a highly effective combined therapy, could increase the solubility and even the bioavailability of traditional compounds. It is expected that systems similar to solid S2 could represent a powerful alternative to overcome antibiotic resistance a in a near future.

## 4. Conclusions

In the scope of the presented study, a new active material based on silica xerogel is prepared from volcanic tuff, which is an inexpensive and sustainable silica precursor. Taking into account the promising textural properties of the silica xerogel such as its mesoporous structure, suitable pore and particle size in addition to its lightness, a ε-poly-l-lysine capped silica xerogel is developed for the first time to enhance linezolid spectrum against Gram-negative bacteria. While free linezolid or linezolid loaded silica xerogel is not active against *E. coli and P. aeruginosa,* a linezolid-loaded silica xerogel capped with ε-poly-l-lysine shows high bactericidal activity for both bacteria. The combination of linezolid and ε-poly-l-lysine on the silica xerogel provide a synergistic effect on the antibacterial activity not only in Gram-negative but also in Gram-positive bacteria such as *S. aureus*. The prepared material S2 showed a 114-, 311- and 40-fold higher efficacy in *E. coli*, *P. aeruginosa* and *S. aureus*, respectively. The results reveal that silica xerogels can be utilized to design capped drug carrier systems and that can be applied in the development of highly effective antibacterial materials. This provides not only a reduction of production cost with using inexpensive carriers but also boost the concept of developing new antibacterial agents against Gram-negative bacteria using already known antimicrobial agents commonly used for Gram-positive bacteria.

## Figures and Tables

**Table 1:** Prepared materials.

Material	Composition
Solid S0	Silica xerogel
Solid S1	Silica xerogel loaded with linezolid
Solid S2	Silica xerogel loaded with linezolid and capped with ε-poly-l-lysine

**Table 2:** Textural properties and bulk density of solid S0.

Material	S_BET_^a^ (m^2^ g^−1^)	Pore Size (nm)	Pore Volume (cm^3^ g^−1^)	Bulk Density (g cm^−3^)
solid S0	195	10	0.50	0.037

^a^ S_BET_ indicates surface area of the silica xerogel.

**Table 3:** Physicochemical properties of the materials.

Material	Zeta Potential (mV)	Electrophoretic Mobility (μm cm V^−1^ S^−1^)	Conductivity (mS cm^−1^)
solid S0	−46.1 ± 0.1	−3.62 ± 0.01	0.0327
solid S1	−42.0 ± 1.1	−3.29 ± 0.09	0.0314
solid S2	16.9 ± 0.6	1.33 ± 0.05	0.0271

**Table 4:** Linezolid and ε-poly-l-lysine content of solid S1 and S2.

Material	Linezolid (mmol g^−1^)	ε-poly-l-lysine (mmol g^−1^)
solid S1	0.188	-
solid S2	0.187	0.022

**Table 5:** Concentration of linezolid and ε-poly-l-lysine (μg mL^−1^) able to reduce until 50% the viability of the bacteria growth (5 × 10^5^ CFU mL^−1^).

Active Compound	*Escherichia coli*	*Pseudomonas aeruginosa*	*Staphylococcus aureus*
linezolid	---	---	1.00
ε-poly-l-lysine	0.13	0.21	0.08

**Table 6:** Concentration of S1, S2 and S3 (μg mL^−1^) able to reduce until 50% the viability of the bacteria growth (5 × 10^5^ CFU mL^−1^).

Material	*Escherichia coli*	*Pseudomonas aeruginosa*	*Staphylococcus aureus*
solid S1	---	---	1.37
solid S2	0.04	0.02	0.07
solid S3	0.29	0.21	0.51
